# Cultural background shapes spatial reference frame proclivity

**DOI:** 10.1038/srep11426

**Published:** 2015-06-15

**Authors:** Caspar Goeke, Suchada Kornpetpanee, Moritz Köster, Andrés B. Fernández-Revelles, Klaus Gramann, Peter König

**Affiliations:** 1 Institute of Cognitive Science, University of Osnabrück, Osnabrück, Germany; 2 College of Research Methodology & Cognitive Science, Burapha University, Chon Buri, Thailand; 3 Center for Cognitive Science, Technical University of Kaiserslautern, Kaiserslautern, Germany; 4 Institute of Psychology, University of Osnabrück, Osnabrück, Germany; 5 Department of Physical Education and Sport, Faculty of Sport Sciences, University of Granada, Granada, Spain; 6 Biological Psychology and Neuroergonomics, Technical University Berlin, Berlin, Germany; 7 Institute for Neural Computation, University of California San Diego, San Diego, USA; 8 Dept. of Neurophysiology and Pathophysiology, University Medical Center Hamburg-Eppendorf, Hamburg, Germany

## Abstract

Spatial navigation is an essential human skill that is influenced by several factors. The present study investigates how gender, age, and cultural background account for differences in reference frame proclivity and performance in a virtual navigation task. Using an online navigation study, we recorded reaction times, error rates (confusion of turning axis), and reference frame proclivity (egocentric vs. allocentric reference frame) of 1823 participants. Reaction times significantly varied with gender and age, but were only marginally influenced by the cultural background of participants. Error rates were in line with these results and exhibited a significant influence of gender and culture, but not age. Participants’ cultural background significantly influenced reference frame selection; the majority of North-Americans preferred an allocentric strategy, while Latin-Americans preferred an egocentric navigation strategy. European and Asian groups were in between these two extremes. Neither the factor of age nor the factor of gender had a direct impact on participants’ navigation strategies. The strong effects of cultural background on navigation strategies without the influence of gender or age underlines the importance of socialized spatial cognitive processes and argues for socio-economic analysis in studies investigating human navigation.

Spatial navigation is a central human cognitive skill. Various scientific studies have concentrated on investigating differences in navigation performance and navigation strategies, in particular dividing egocentric and allocentric navigation. Navigation can be based on different reference frames using distinct coordinate systems to encode spatial information. An egocentric coordinate system is located within the agent and is conditioned upon his orientation in space, while an allocentric coordinate system codes relations between objects, independent of the observers’ orientation^1^. Several studies in spatial navigation demonstrated pronounced changes in spatial navigation performance in elderly participants^2^. Comparisons of navigation performance in young and elderly participants revealed an age-based decline in speed and accuracy as well as changes in navigation strategy^3^ and their underlying preference for using an egocentric or allocentric spatial reference frame. Changes in preference that lead to using an egocentric reference frame with older age might be directly related to changes in the neural substrate subserving computation of different reference frames. However, individual proclivities to use an egocentric or an allocentric reference frame can be observed already in young and middle-aged participants^4^,^5^. Considering such pronounced individual differences in the use of an egocentric or an allocentric reference frame, it is important to delineate the underlying mechanisms that shape the proclivity to use different reference frames. In this respect, Gramann and colleagues^6^ revealed specific cortical activation patterns to be associated with the use of particular reference frames. Gramann^7^ argues that both types of reference frames (egocentric and allocentric) are soft-wired (genetically predetermined) on a neuronal level, but that several additional factors influence the development of individual reference frame proclivities. A rough separation can be made between environmental and biological factors. On the one hand, the development of the physical structure (i.e., maturation) and motor skills (e.g. upright walking) impacts the neural circuitry underlying computation of a distinct reference frame. On the other hand, cultural factors like socioeconomic status, language, or urbanization strongly influence maturation of individual spatial reference frame proclivities^8^,^9^,^10^. This view is in line with other studies investigating individual differences in spatial cognitive abilities. Waller^11^ showed that gender, computer and navigation interface proficiency shape individual differences in navigation performance. Hegarty and colleagues^12^ performed structural equation modelling comparing a variety of spatial tasks and learning strategies, confirming that gender differences were present throughout most tasks that were considered. More recently, Wolbers & Hegarty^13^ summarized existing literature regarding individual differences in spatial cognition and concluded that human spatial navigation is influenced by a multitude of factors, including gender and age, but also the individual preference to use different environmental cues. In this light, the current study further investigates the underlying mechanisms shaping individual preferences of reference frame use and navigation performance. We examine both, nature and nurture-based factors, specifically, gender, age, and cultural backgrounds, and some of their interactions to develop a more complete picture of individual differences in spatial performance and reference frame proclivities.

## Gender Differences in Spatial Cognition

Over the last decades, some studies have shown that males outperform females in specific spatial tasks^14^,^15^. Among the most prominent task examples are the Morris Water Maze^16^ and the Mental Rotation task^17^. In both tasks, males make fewer errors and take less time to solve the task. Furthermore, several studies reported that females are less likely to use an allocentric navigation strategy^18^,^19^,^20^. In an attempt to explain the origin of a potential male superior performance in spatial navigation, Perdue and colleagues^21^ reviewed several studies to conclude that the range-size hypothesis fits best with the observed data in carnivores. This hypothesis claims that males need to travel larger distances to mate with more females. Roof and colleagues^22^ investigated the biological basis of sex differences in spatial navigation and demonstrated that the “male-hormone” testosterone improves spatial navigation performance in females^23^. The above cited studies emphasize the role of biological differences to account for the observed sex differences in spatial navigation. In contrast to such an approach, Saucier and colleagues^24^ suggested that differences between men and women simply reflect differences in the underlying navigational strategy. In line with such an explanation, Grön and colleagues^25^ described differences in cortical areas activated in men and women during navigation, pointing to different navigation strategies. Finally, Hoffmann and colleagues^10^ concluded that differences in navigation performance or strategy use between males and females are not based on genetic differences but rather on differences in social hierarchies and educational standards. Generally, the degree and characteristics of gender and social influences on spatial cognition and navigation are still under debate.

## Developmental & Aging Aspects of Navigation

The effects of maturation and aging on navigation have gained attention by many researches in the field. The development of brain structures central for spatial cognitive processes (i.e. the hippocampus) already start prenatally and, compared to other regions of the brain, seems to happen rather early^26^. During childhood, several milestones like crawling, upright walking, and the ability to perform head movements are crucially important for the maturation of neural structures^27^, which are in turn important for the development of spatial strategies. Besides those early developmental aspects of navigation, lifespan developments have a strong impact on navigation. Most studies found strong evidence of a decline in spatial navigation performance with increasing age^28^,^29^,^30^,^31^. Moffat and colleagues^32^ showed that performance deficits in elderly participants correlated with decreased activation in hippocampal and parahippocampal areas during virtual navigation. Holdstock and colleagues^33^ performed navigation tasks with patients that suffered from selective hippocampal damage and demonstrated that the loss of hippocampal tissue was associated with the inability to successfully employ allocentric navigation strategies. Similarly, Iaria and colleagues^34^ investigated how aging affected the use of particular navigation strategies and corresponding performance. Their results suggest that elderly participants have more difficulties in using allocentric navigation strategies compared to younger participants. In summary, most researchers agree that navigation performance decreases with age and that distinct brain regions are important for the use of egocentric and allocentric strategies^35^,^36^. However the exact relations of changes in neural networks with performance decline in spatial tasks are still widely discussed.

## Cultural Influences on Navigation

Over the last decades, cross-cultural research has revealed astonishing differences that have had strong implications in the field of sociology and psychology. One of the most investigated factors was the distinction between individualism vs. collectivism. Markus and Kitayama^37^ showed that people from Asian and Western cultures have “*different construal’s of one self, of others and the interaction of the two.*” They demonstrated that Asian cultures tend to see themselves and others as more interconnected than people in Western cultures and that people from Asian countries try more to fit into a society, while people from Western cultures aim to emphasize their uniqueness and independence. Masuda and Nisbett^38^ compared reports about scene perception between Eastern and Western cultures and showed that Eastern people talked more about global scene information, the background, and the environment, while Westerners reported more about single salient objects in a scene. Similarly, Chua and colleagues^39^ demonstrated that such different verbal reports were accompanied by different viewing behaviors; American participants fixated longer on the center of the screen while the Chinese participants made more saccades towards the background. Majid and colleagues^40^ showed that the way participants expressed spatial relations varies between cultures. Haun and colleagues^9^ took this idea a step further and demonstrated a correlation between the dominant linguistic spatial reference frame and the dominant reference frame applied in a spatial memory task. Lovett and Forbus^41^ applied a different approach by using a Structure Mapping Engine to model observed differences in spatial problem solving performances between North Americans and the Munduruku, an indigenous South American tribe. In summary, investigations on the cultural background demonstrate an impact of culture on many aspects of human behavior and cognition.

## Current Study

As described above, there are several factors that influence navigation and spatial cognition in general. Here we investigated how well the factors gender, age, and cultural background can account for individual differences in navigation performance and spatial reference frame proclivity. Specifically, we tested these different factors as predictors. Previous studies have focused on studying these factors mostly in isolation, sometimes by a combination of two factors. To our knowledge, there are very few studies that provide results about all three factors regarding spatial cognition. As a task, we applied a VR star-field path integration task including horizontal (yaw) and vertical (pitch) rotation changes. This paradigm is based on the tunnel task^5^,^42^ that reliably demonstrated individual differences in reference frame proclivities in virtual navigation with turns in the yaw axis only. While all participants perceive the same visual information in the tunnel task^43^, different cortical activation patterns were observed for participants using an allocentric or an egocentric reference frame during navigation^6^,^44^,^45^. This paradigm was adapted to spatial navigation in 3D environments, replicating previous results^46^. Establishing an online version of the 3D spatial reference frame proclivity task, Goeke and colleagues^47^ showed that 207 out of 260 participants could be assigned to either the Turner group, preferring an egocentric reference frame, or the Nonturner group, preferring an allocentric reference frame for navigation. Using the same paradigm as Goeke and colleagues, we collected additional data and analyzed reference frame proclivities and performance data from 1823 participants worldwide. For performance analysis we employed two measures, median reaction time and the amount of incorrect responses for each participant. Most importantly, spatial reference frame proclivity was assessed by analyzing the behavioral responses. Altogether this study aimed to reveal the effects and interdependencies of the most important factors regarding spatial cognition within the general population.

## Results

### Reaction Time

**Figure 1:**
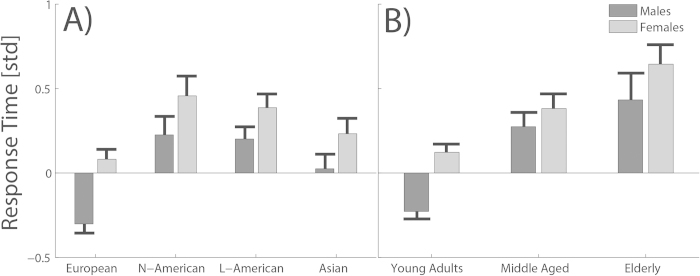
Reaction times for the different subgroup populations. The y-axis shows the mean reaction time in normalized standard deviations. The x-axis separates the different subgroups. Panel **A** shows the reaction times for the different cultural groups, subdivided into males (dark grey bars) and females (light grey bars). Panel **B** shows the reaction times for the three different age groups again separated by the gender factor. The error bars illustrate the standard error of the mean.

After an initial cleaning and screening for individuals’ spatial reference frame proclivity (see Methods) we first analyzed participants’ performance with respect to the independent factors gender, age, and cultural background. Rank transformed and normalized reaction times revealed clear differences for both factors, gender and age (Fig. 1B). Smaller variations were observed for different cultures (Fig. 1A). Accordingly, a three way ANOVA demonstrated significant main effects for the factors gender (F(1,1147) = 5.17, p = .023, partial η^2^ = .005) and age (F(2,1103) = 9.00, p < .001, partial η^2^ = .016), and a borderline effect for the factor culture (F(3,1103) = 2.59, p = .052, η^2^ = .007). None of the two interaction terms considered reached significance. The partial eta squared indicated that age had the strongest impact on the observed variations. Surprisingly, gender had a significant, but weaker influence. Post hoc pairwise comparisons showed that males reacted significantly faster than females (p < .05) and that young participants had significantly shorter reaction times compared to middle-aged (p < .01) and elderly participants (p < .01). Elderly participants had the slowest reaction times but the difference between these participants and middle-aged participants was not significant (p > .1). Although overall culture had only a borderline effect, post hoc pairwise comparisons revealed that European participants reacted faster than participants in all other cultural groups North Americans (p < .01), Latin Americans (p < .01), and Asians (p < .01). North Americans reacted slowest, but differences to other cultural groups were not significant.

### Error Rate

**Figure 2:**
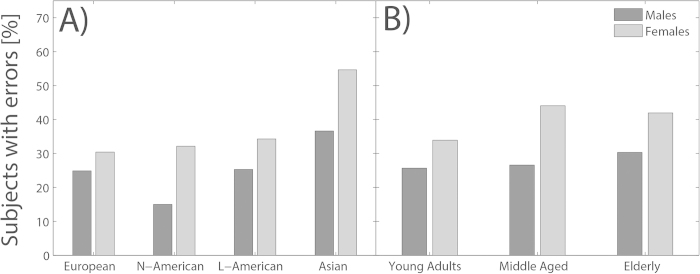
Error rate in the subgroup population. The y-axis indicates the percentage of participants who made at least one (or more) error(s) during the experiment. The x-axis divides the participants into different subgroups. Panel **A** shows the error rates for the four cultural groups, separately for males (dark grey bars) and females (light grey bars). Panel **B** depicts the error rate for the three age groups, again separately for males and females.

Analysis of error rates showed a significant influence of the factor gender (Wald *χ*^2^ (1) = 12.239, p < .001) as well as of the factor culture (Wald *χ*^2^ (3) = 27.051, p < .001). Figure 2 shows that the proportion of subjects who made at least one error was significantly higher for the female population (light grey bars) compared to males (dark grey bars). Panel A of Fig. 2 reveals that Asians were significantly more likely to make errors compared to the European reference group (Wald *χ*^2^ (1) = 23.682, p < .001). Contrasts from other cultural groups did not reach significance. Interestingly, the proportion of subjects who made one or more errors did roughly stay constant with age (Fig. 2B), such that no significant age effect was observed (Wald *χ*^2^ (1) = 1.426, p = .232).

### Spatial Reference Frame Proclivity

**Figure 3:**
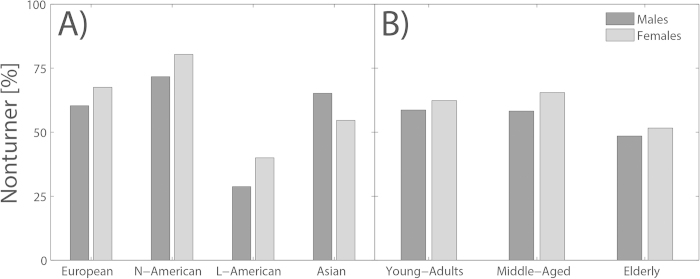
Reference frame proclivities of the subgroup populations. The y-axis shows the percent of Nonturners (compared to Turnesr) for a given subgroup. The x-axis separates the subgroups by their cultural background (Panel **A**) and age (Panel **B**). The data is again subdivided between males (dark grey bars) and females (light grey bars). The height of the bars illustrates the relative amount of Nonturners in the respective subgroup.

To investigate the impact of gender, age, and cultural background on reference frame proclivity, we analyzed the type of reference frame (egocentric vs. allocentric) preferred by participants in the navigation task. Only the factor culture revealed a significant effect (Wald *χ*^2^ (3) = 54.874, p < .001). Figure 3A illustrates the ratio of Nonturner (allocentric) participants for the four different cultural groups, separately for both genders. The most prominent difference can be observed between North American and Latin American populations. While North Americans strongly preferred an allocentric reference frame, Latin Americans clearly preferred an egocentric reference frame. Accordingly, the regression results demonstrated that North-Americans more often used an allocentric reference frame than Europeans (Wald *χ*^2^ (1) = 7.106, p = .008) while Latin Americans significantly less often used an allocentric reference frame compared to Europeans (Wald *χ*^2^ (1) = 41.416, p < .001). Asians did not differ from the European group (Wald *χ*^2^ (1) = 0.467, p = .495). The factor of gender did not reach significance (Wald *χ*^2^ (1) = 2.477, p = 0.116), but the interaction of culture and gender became borderline significant (Wald *χ*^2^ (3) = 7.456, p = .059). A closer look revealed a significant gender-culture based deviation only for the Asian population (Wald *χ*^2^ (1) = 7.284, p = .007). This interaction was driven by the fact that females (Fig. 3A, light grey bars) more often used the allocentric reference frame compared to males (Fig. 3A, dark grey bars) in European, North and Latin American populations. However, this principle was reversed in the Asian population; Asian males were more often Nonturner than Asian females. Fig. 3 B shows the differences in spatial reference frame proclivity in the three age groups. Although a small trend towards an egocentric preference in elderly subjects was observed, the factor age (Wald *χ*^2^ (2) = 2.714, p = .099) did not have a significant influence on reference frame use.

## Discussion

We employed a virtual path integration paradigm with which we recorded data from 1823 participants over the course of about two and a half years. In comparison, only very few studies in the field of spatial navigation have attempted to develop a task that targets such a large overall population, but typically only recruited between ten and fifty participants. A larger database allows for investigation of multiple dependencies and underlines the ecological validity of our results. With this approach we could analyze the influence of gender, age, cultural background and some of their interactions on navigation performance and reference frame proclivity. To our knowledge the current study is the first to provide results with respect to all three factors individually and some of their interactions. Our results demonstrate that gender, age as well as cultural background play, to varying degrees, a role in individual reference frame proclivities.

Overall, the question remains to what extent the reported findings can be generalized and to what degree our results match with other studies investigating navigation. The reference frame proclivity test at hand provides a quite specific and in some way minimalistic approach to studying individual differences in spatial cognition. However, similar to our design, many studies of spatial navigation use virtual environments that only provide visual flow as specific input to derive changes in position and orientation. Here the natural change of idiothetic input during navigation is absent, which might lead to changes in orientation behavior. This was demonstrated by Klatzky and colleagues^48^ as well as Chance and colleagues^49^, clearly showing decreased homing performance after path integration with decreasing idiothetic information. Hence, one could speculate that the observed differences in reference frame proclivity might only have been evoked because of the inherent sensory mismatch between visual and proprioceptive information in our study. Indeed, Ehinger and colleagues^50^ demonstrated differential cortical activation in an active, with vestibular and proprioceptive stimulation, vs. passive triangle completion task. Furthermore, earlier studies^6^ investigating the tunnel paradigm reveled differential activations of cortical and subcortical structures for egocentric and allocentric reference frame use, indicating different navigational networks to be used by different strategy groups. In addition, Gramann and colleagues^6^,^44^,^45^ demonstrated increases in frontal theta during more difficult aspects of virtual path integration tasks. This is in line with results from other virtual navigation tasks like the one used by Kahana^51^ and colleagues who implemented a more complex virtual navigation setup and recorded oscillatory subdural activity on epilepsy patients. Interestingly, they found an increase in spectral theta power during recall of spatial information. Similarly Bischof & Boulanger^52^ reported a correlation of theta band power and task difficulty during maze navigation. Altogether these results indicate that insights gained on the basis of virtual navigation paradigms reflect meaningful and highly relevant features in spatial cognitive processes.

As indicated by both ANOVA results and corresponding effect-size analysis the factor age had the strongest influence on reaction times. This is in line with Moffat and colleagues^31^ who found that elderly participants reacted slowest during a navigation task compared to younger participants. However, opposite to earlier findings, we observed an approximately constant error rate across different ages. A possible explanation for this is that the age range of elderly participant included subjects 50 years of age and older, with an average age of about 60 years with heterogeneous subgroups. Indeed, many other studies have tested elderly participants that were ten to twenty years older than participants in the present study. Thus, a direct comparison of the present results with other studies on age-related changes in navigation should be treated with care, and a larger sample of older participants aging 70 and older might potentially show a stronger age effect. Furthermore, it is noteworthy to mention that erroneous responses might have happened due to a variety of reasons. Individual high error rates lead to the assumption that a particular subject has rather poor spatial cognitive abilities. However some participants might have had troubles understanding the task demands, a flawed visual perception and/or a wrong interpretation of the arrows’ pointing direction. All these factors could also have led to an increase of error rate. Finally very few (<1%) subjects had extremely high error rates, which might have been present due to distraction or lack of attention during the task.

As expected, the factor gender significantly influenced both reaction times and error rates. Males responded faster and made fewer errors than females. Such a male advantage is in line with several previous studies^14^,^15^,^16^,^17^. Interestingly, this male advantage was present throughout all cultures and age groups for both types of performance measures. The absence of performance-based interaction effects suggests that the observed male advantage in this spatial navigation task is a rather general phenomenon and not limited to a certain culture or age group. One reason for the male superiority in spatial performance might be more training, in particular with virtual (computer based) navigation. Several studies have shown that playing computer games improves mental rotation skills^53^ and pointing accuracy^54^. Furthermore, social expectancy and task anxiety might have had a strong impact of the observed gender differences. Moè & Pazzagli^55^ found that women have a lower expectancy in spatial task performance and that this lowered confidence, causing worse performance during navigation^56^,^57^. Lawton and colleagues^58^ showed that women demonstrate higher anxiety levels during such tasks, which also reduced task performance^59^. In summary, several factors contribute to the overall lower spatial performance observed in the female group. However, the additional influence of cultural background on navigation performance is compelling. In particular, European participants reacted quicker than anyone else and Asian participants made significantly more errors than other cultural groups. However, more detailed investigation regarding socio-economic influences must be carried out to understand the underlying mechanisms of such effects. In essence, the results of our performance measures are compatible with the literature in the field and extend and attribute an influence of all three factors on performance measures.

Unexpectedly, the analysis of spatial reference frame proclivity revealed a significant effect of cultural background only, while there was no main effect of the factors gender or age. Particularly surprising is that gender factored in only by an interaction with cultural background. The majority of earlier studies reported that females preferred egocentric strategies over allocentric strategies^18^,^19^,^20^,^21^. Our results argue against such conclusions and suggest that gender differences in spatial reference frame proclivity are dependent on the cultural background of participants. In particular, our findings suggest that Asian males use an allocentric reference frame more often than Asian females, while for European, North and Latin American populations such a gender effect is reversed. The hypothetical decline of an allocentric navigation reference frame with increasing age was only slightly visible in our data. However, sampling in the elderly groups was rather poor and heterogenic. In the future, a denser sampling for elderly participants will be pursued to investigate this question. Most surprising was the particularly strong influence of cultural background on spatial reference frame proclivity. Here, it is worth mentioning that the definition of egocentric and allocentric reference frames as we used them in our study, also correspond to the definition of reference frames in a linguistic sense. Levinson’s abstraction of spatial frames of reference (FoR) describes three different forms of reference frames^40^,^60^,^61^. The ‘Relative FoR’ uses a coordinate system that is aligned with the cognizing subject and can be roughly compared to an egocentric reference frame used in our study. The second FoR describes the ‘Absolute FoR’ that uses fixed bearings like magnetic north, which is again highly similar to the definition or our allocentric reference frame. The last FoR according to Levinson is the ‘Intrinsic FoR’ that provides coordinates centered within an object that provides canonical orientation (e.g., a car with a defined front and rear end). However, for large-scale spatial tasks with no unique other objects involved the intrinsic FoR might be not as useful as compared to small scale tasks (e.g. table-top arrangements) because objects might be too large or far away to be clearly recognized or to provide useful object orientation information. In general, an allocentric reference frame was used more frequently in all cultures except the Latin American culture. However, strategies did not vary along the dimensions commonly discussed in cross-cultural psychology, i.e., Western vs. Eastern populations^37^,^38^,^39^. This supports the idea that culturally related preferences in spatial reference frame proclivities are generated by other mechanisms than those investigated by most cross-cultural studies. In particular, the concept of collectivism vs. individualism seems to not be an adequate model for explaining the observed effects. However, both North and Latin American cultures significantly differed from the European reference group. The fact that these findings are equally present in both males and females supports the validity of the observed effect. Nevertheless, the characteristics of the observed differences are not fully understood. Why do Latin Americans overall prefer an egocentric and North Americans an allocentric reference frame? Brown and Levinson^62^ investigated the spatial language use of the Mayan language Tzeltal. They reported that both groups avoid egocentric terms in their language compared to English speaking people. Similarly, Levinson^63^ showed that the Guugu Yimithirr, an Australian aboriginal tribe, describe spatial relations using absolute or allocentric reference systems compared to people from Western languages who use egocentric coordinates (left, right). On the first, view it might be tempting to relate the Latin American population in our study to the indigenous populations of Brown and Levinson; however, the direction of the observed effect is reversed. Moreover, in contrast to those indigenous people the vast majority of participants in our study, including the ones from Latin America were urbanized and in one way or another related to a university context. Hence the daily navigation scenery of both the Latin and the North American group was rather comparable. An alternative interpretation is related to the socio-economic factors that vary across different culture groups. As Hoffman and colleagues^10^ reported, variations in educational standards and social hierarchies could account for observed differences in spatial performance between males and females. In fact, several underlying socio-economic variables might have generated such culture effects and future studies need to investigate this in more detail.

## Methods

### Online Navigation Task

For the purpose of present study, we translated the web page (http://www.navigationexperiments.com/TurningStudy.html) into 10 different languages (German, English, French, Spanish, Portuguese, Russian, Turkish, Chinese, Thai, and Korean) and established cooperation between different research groups, which all advertised the study locally and recruited participants with varying ages and genders. A back translation validated that the instructions and questionnaire were correctly translated between the different languages. Overall, we followed two approaches during data collection. Most importantly, we aimed to obtain a high number of participants for each age, gender and cultural subgroup, in order to allow proper statistics. Second, we targeted the general audience and tried to reach as many subjects as possible. All of the participants performed the experiment independently with detailed instructions given during the procedure. All of the subjects were informed about the purpose of the study, and informed consent was obtained from all of the subjects during the procedure. Data recording was carried out in accordance with the approved ethics guidelines of the University of Osnabrück. The experimental protocols were approved by the Ethics Committee of the University of Osnabrück.

**Figure 4:**
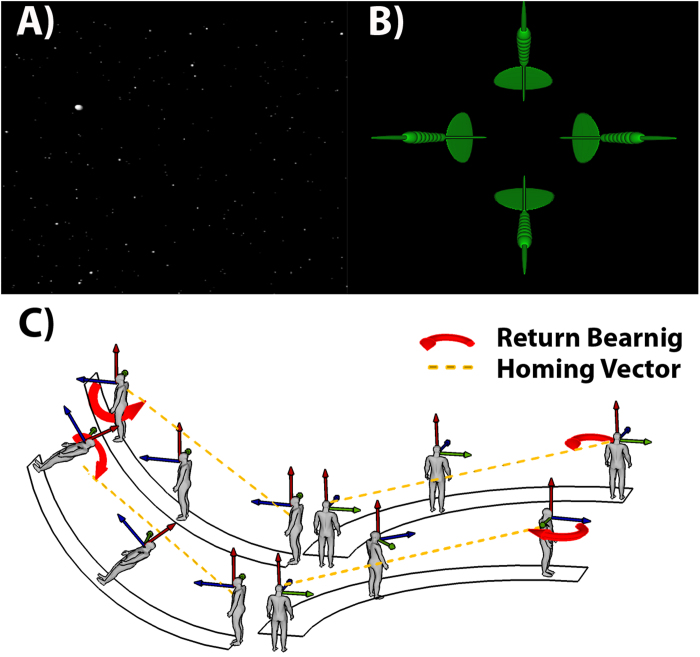
Experimental paradigm. **A**) Screenshot of the star-field passage in the online navigation experiment. During the passage, the white dots (stars) induced visual flow, indicating a turn into one direction. **B**) Forced choice arrow selection in the experiment after a 60-degree, rightward turn. The arrow pointing to the right indicates the homing vector in line with an egocentric reference frame; the arrow pointing to the left is congruent with an underlying allocentric reference frame. Choosing one of the other two arrows (up and down in this case) was counted as an incorrect response. **C**) Turner and Nonturner responses, respectively in a schematic 3D drawing. The two upper drawings display the spatial reference frame proclivity for a Nonturner (allocentric) navigator during the turning segment. Most importantly, Nonturners do not change (update) their cognitive heading during the turning segment. The upper-left drawing shows the Nonturner spatial reference frame proclivity on a pitch trial, while the upper-right drawing shows a yaw trial. The yellow path indicates the homing vector back to the starting location. The red, curved arrow displays the return bearing at the end of the turning segment. The two lower drawings show a Turner (egocentric) navigator during pitch (left) and yaw (right) navigation. Importantly, the Turner updates his/her cognitive heading during the turning segment and therefore has a return bearing (red arrow) that is mirror-reversed compared to the Nonturner’s bearing.

As a navigation task, we used the tunnel paradigm, originally proposed by Gramann and colleagues^5^, as it provides a fast and clear categorization of the preferred spatial reference frame. The task of the participants in this particular paradigm was to choose one out of 4 homing arrows (Fig. 4B) that indicates the way back to the starting position (homing arrow) after a visually presented path (Fig. 4A). Each path was constructed such that an initial straight segment was followed by a turning segment (left, right, up or down). Three different turns were used with 30, 60, and 90 degrees. After every stimulus turn was another straight segment. All of the segments were visible for about three seconds and smoothly transitioned into one another. The combination of each direction (4) and angle (3) was rendered as a separate video in “mp4” format and displayed using Flow Player®. Each video was shown twice in a randomized order, such that all of the participants performed 24 trials. Questionnaire data were retrieved after the tunnel task. The participants could use either an egocentric (Turner) or an allocentric (Nonturner) reference frame (see Fig. 4C), associated with distinct homing arrows. The essential difference between both strategies is based on whether the participants updated their cognitive heading along with the stimulus turn seen on the screen. In the lower panels of Fig. 4C the human navigator changes (updates) her cognitive heading direction according to the heading changes indicated through changes in visual flow. As a consequence, she chooses an arrow pointing “up and back” after passage with a turn upwards and “right and back” after a passage with a turn to the right. Reversely, the other two drawings show a human navigator who does not update her heading according to visual flow changes during the stimulus turn. Therefore, the navigator points “down and back” after a passage with a turn upwards and “left and back” after a passage with a turn to the right. From here on, we call the first type of reference frame users “Turner” or egocentric and the second type of reference frame users “Nonturner” or allocentric. There were two additional arrows pointing into wrong directions (i.e., to the left or right after a passage up or down, or up and down after a passage to the left or the right) serving as catch responses (erroneous responses).

### Participants

Over the course of 34 month, 1823 participants performed the online experiment. We excluded all participants with incomplete or ambiguous (e.g. naming no or more than one country for cultural background) data, teenagers below the age of 18, and people from those geographic regions with sparse sampling (see Cleaning & Clustering section). Consequently, 372 participants were removed from the dataset and the remaining 1451 participants were used for analysis. Out of these, 872 (60.10%) were males and 579 (39.90%) were females. The grand average age of the participants was 26.23 years (SD = 11.47). Overall, we were able to recruit participants from over 30 countries and 4 continents. Although the participant distribution was not equal across all of those countries, each of our cooperation partners in Spain, Thailand, Mexico, and the U.S. recruited at least 150 participants. As the study was advertised by various research institutes in an academic context, most participants were related to a university context. In total, 205 of all 1451 participants were left handed. No participant received reimbursement for the experiment, but all of the participants were offered information on their preferred reference frame at the end of the experiment.

### Determining Reference Frame Proclivities

**Figure 5:**
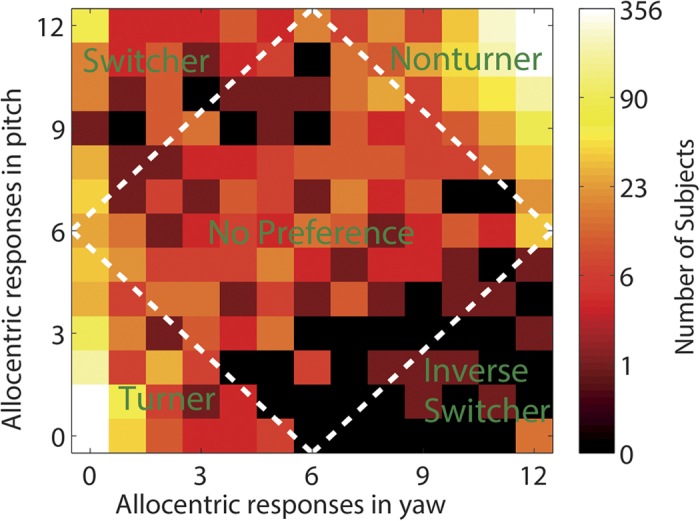
Classification of all subjects into reference frame strategy groups. The y-axis indicates the number of allocentric responses in pitch, the x-axes indicates the number of allocentric responses in yaw. The color of the squares reflects the number of subjects with identical ratios of allocentric vs. egocentric responses for both yaw and pitch (bright yellow indicates many subjects; dark brown indicate few subjects). The logarithmic scale to the right shows the number of subjects corresponding to each color, with rounded values. The white, dashed line marks the boundaries between the reference frame strategy groups. Additionally, the labels (in green) indicate the names of the respective strategy groups.

The spatial reference frame proclivity categorization was performed in accordance to previous studies. In short, we categorized all subjects according to their ratio of allocentric and egocentric responses (not taking erroneous responses into account) into one out of five classes (Turner, Nonturner, Switcher, Inverse Switcher and No Preference). Figure 5 illustrates this classification. The Participants who used the allocentric response in at least 75% of all cases were classified as Nonturners (upper-right corner), while the participants using the egocentric reference frame in at least 75% of cases were classified as Turners (lower-left corner). Switchers and Inverse Switchers changed reference frame use depending on the axis of rotation, while the “No Preference” participants randomly switched between the reference frames. For more information about the category labeling, category boundaries and parameters used, see and Goeke and colleagues^47^.

### Cleaning & Clustering

We needed to ensure that the sample size of each investigated subgroup was large enough to be able to perform valid statistical tests. Two restrictions had to be made due to this statistical consideration. First, we could not investigate all of the interactions among our independent factors. Second, the participants who were too young, came from a geographic region with very few participants, or did not have a clear proclivity for a spatial reference frame (Turner or Nonturner) were excluded from the data set. Specifically, we were only able to recruit 5 participants from Latin America aged 50 years or older. As a consequence, we decided to not include the 2-way-interaction of culture and age or the 3-way interaction of culture, age and gender. Instead, for all of the subsequent analyses, we only considered the main effects of age, gender, and culture; the 2-way interaction of gender and culture; and the 2-way interaction of gender and age.

The gender analysis was simplest using the dichotomous response from the participants. Cultural categorization was based on the question: “In which country did you grow up”. However, again considering the sample sizes, we were not able to analyze each country individually. Hence, we decided to group all of the countries with respect to geographical closeness, which is one of the most intuitive ways to coarsely separate different cultures. As a result, we assigned all participants to one out of four cultural groups (Europe, North America, Latin America and Asia). The remaining participants with cultural backgrounds other than those mentioned above were removed from the data set because their total sample sizes were too small for further statistical analysis. Regarding age, we first excluded all of the participants who were younger than 18, as we could not ensure that those participants had performed the study independently by themselves. Then, we divided the participants into one out of three age-groups: young adult, middle-aged and elderly participants (young-adult: range = 18–30 years, mean = 21.54 years, SD = 3.52 years; middle-aged: range = 31-50 years, mean = 37.05 years, SD = 5.69 years; elderly: range >50 years, mean = 60.53 years, SD = 10.54 years).

Finally, we also needed to ensure a rather homogenous distribution of our categorical dependent variable, namely spatial reference frame proclivity. Overall, the subjects were assigned to one out of five possible strategy classes. However, the group sizes of participants other than Turner or Nonturner were relatively small and could not be statistically analyzed after in combining our independent factors (e.g. young female Switchers from Asia), again due to too small sample sizes. Consequently, the data analyses focused only on the distribution of the two main reference frame strategy groups; hence we removed the participants who followed a different minor strategy. In total we removed 372 subjects from the data set, leaving 1148 subjects for the consequent analyses of spatial reference frame proclivity and performance. As a result, each investigated subgroup (see Table 1 and Table 2) had at least 30 participants, which ensured valid statistics.

### Analysis

**Figure 6:**
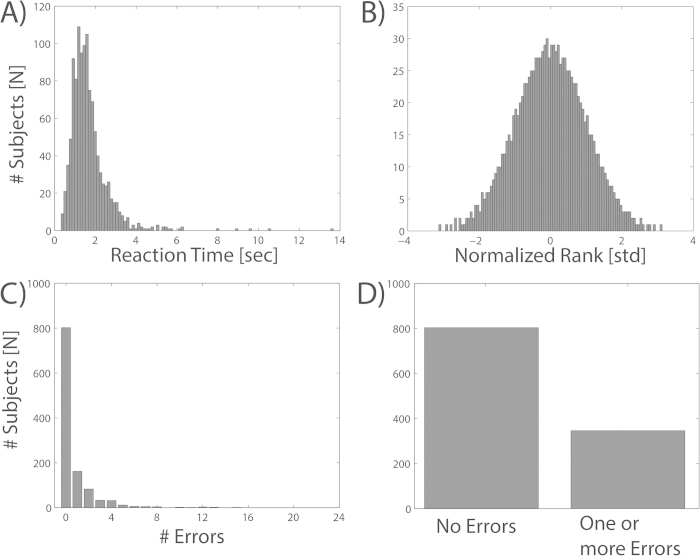
Distribution properties of the performance measures. Panel **A** shows the distribution of median reaction times for all of the participants. Panel **B** illustrates the distribution of reaction times after a normalized rank transformation. Panel **C** shows the histogram of errors for all of the participants. Panel **D** illustrates the distribution of participants into the two error groups (no errors, one or more errors).

For the analysis of reaction time, we first calculated the median response latency for each participant. The resulting distribution of response times over subjects was not normally distributed; rather it was slightly skewed in the positive direction meaning that most of the participants reacted rather quickly and only a few participants reacted slowly (Fig. 6A). Hence, in a second step we rank transformed reaction time data and mapped the resulting values to a Gauss distribution with mean 0 and standard deviation 1 to reach a normal distribution (Fig. 6B). Having fulfilled the normality requirement, we employed an ANOVA with reaction time as a dependent variable and age, gender and cultural background as fixed effects and independent factors. Finally, we used Turkey’s honest significant difference (HSD) test to compare the individual differences between the various subgroups.

The second measure of navigation performance was defined as the amount of incorrect responses by each participant. We counted an error if a participant chose an arrow pointing horizontally after a vertical passage or vice versa, as such a behavior indicates a confusion of the yaw and pitch axes. However, most of the participants did not make any errors (Fig. 6C). Hence, calculating the average number of errors suffered immensely from outliers (subjects with 5 or more errors) and thus violated the homogeneity of variance assumption necessary to perform a parametric test. In order to still test for differences regarding error rate, we transformed the data into a binary variable: participants with one or more errors vs. participants without errors (Fig. 6D). After this transformation, we employed a binary logistic regression with gender and culture as categorical predictors, age as an ordinal predictor, and “error-group” (0 or 1) as the binary response variable. We used a stepwise approach (Wald forward) to find the model that best fitted the data. As a reference category, we chose the most common groups for each investigated factor (Males, Young Adults and European) in order to avoid false discoveries due to data sparseness.

The focus of the reference frame investigation was based on the two dominating reference frame strategies in the current paradigm (Turner vs. Nonturner). There were 686 Nonturner and 462 Turner in our filtered sample data, resulting in a baseline ratio of 59.76% Nonturners. We hypothesized that this ratio of Turner vs. Nonturner participants varied significantly between males and females, different age groups, and culture subpopulations in our dataset. As we intended to use the spatial reference frame proclivity as the dependent variable, we decided to perform a binary logistic regression, similarly to the analysis of error rate. Again, gender and cultural background were used as categorical predictors, while age was used as an ordinal predictor. We used the participants’ spatial reference frame proclivity (0 = Turner, 1 = Nonturner) as the binary response variable. We used the same reference categories (Males, Young Adults and European) as in the error rate analysis and again applied a stepwise (Wald forward) approach.

## Additional Information

**How to cite this article**: Goeke, C. *et al.* Cultural background shapes spatial reference frame proclivity. *Sci. Rep.* **5**, 11426; doi: 10.1038/srep11426 (2015).

## Figures and Tables

**Table 1:** Gender-cultural participant distribution.

Subgroup / Strategy	Males				Females				∑
	European	N-American	L-American	Asian	European	N-American	L-American	Asian	
Nonturner & Turner	426 29.36%	60 4.14%	87 6.00%	112 7.72%	240 16.54%	56 3.86%	70 4.82%	97 6.69%	1148 79.12%
Other Strategies	110 7.58%	16 1.10%	18 1.24%	43 2.96%	67 4.62%	4 0.28%	8 0.55%	37 2.55%	303 20.88%
∑	536 36.94%	76 5.10%	105 7.24%	155 10.68%	307 21.16%	60 4.14%	78 5.38%	134 9.24%	1451 100%

The table shows the total number and fraction of Turner plus Nonturner participants (second row), compared to subjects using a minor reference frame strategy (third row). The last row illustrates the sum of all reference frame strategy groups. Columns two to nine separate the subjects according to their gender and cultural background. The four left columns show the number of male subjects having a European (2), North-American (3), Latin-American (4) and Asian (5) cultural backgrounds, while the four right columns are for female subjects, respectively. The last column sums up the culture and gender groups.

**Table 2:** Gender-age participant distribution.

Subgroup / Strategy	Males			Females			∑
	Young Adults	Middle-Aged	Elderly	Young Adults	Middle-Aged	Elderly	
Nonturner & Turner	573 39.49%	79 5.44%	33 2.27%	348 23.98%	84 5.79%	31 2.14%	1148 79.12%
Other Strategies	155 10.68%	16 1.10%	16 1.10%	78 5.38%	24 1.65%	14 0.96%	303 20.88%
∑	728 50.17%	95 6.55%	49 3.38%	426 29.36%	108 7.44%	45 3.10%	1451 100%

The table shows the total number and fraction of Turner plus Nonturner participants (second row) compared to the subjects using a minor reference frame strategy (third row). The last row illustrates the sum of all reference frame strategy groups. Columns two to seven separate the subjects according to their gender and age. The three left columns indicate the number of young males (2), middle-aged males (3) and elderly males (4). The three right columns show the same for young females (5), middle-aged females (6) and elderly females (7), respectively. The last column sums up the gender and age groups.Legend of Tables.
